# Incense and ritual plant use in Southwest China: A case study among the Bai in Shaxi

**DOI:** 10.1186/1746-4269-7-43

**Published:** 2011-12-13

**Authors:** Peter O Staub, Matthias S Geck, Caroline S Weckerle

**Affiliations:** 1 Institute of Systematic Botany, University of Zurich, Zollikerstrasse 107, 8008 Zurich, Switzerland

**Keywords:** Ethnobotany, Incense, Plant-derived smoke, Ritual plants, Traditional Medicine, Yunnan

## Abstract

**Background:**

Ritual and religious uses of plant-derived smoke are widespread throughout the world. Our research focuses on Southwest China, where the use of incense is very common. This study aims to document and analyze contemporary ritual plant uses by the Bai people of Shaxi Township (Jianchuan County, Dali Prefecture, Yunnan Province), including their related ethnobotanical knowledge, practices, and beliefs.

**Methods:**

The present study builds on previous ethnobotanical research in Shaxi, which started in 2005. Interviews focusing on ritual plant use and associated beliefs were carried out with a total of 44 Bai informants in September 2009 and May and June 2010. The results are supplemented with information on the local religion collected from June to December 2010. All documented species were vouchered, and are deposited at the herbaria of Kunming Institute of Botany (KUN) and the University of Zurich (Z/ZT).

**Results:**

A total of 17 species have been documented for use in incense. They are always used in mixtures and are either burned in the form of powders in a censer or as joss sticks. The smell of the smoke is the main criterion for the selection of the incense plants. Incense is burned for communication with spiritual entities at graves, temples, and cooking stoves, as well as for personal well-being. *Cupressus funebris *Endl., *Gaultheria fragrantissima *Wall., and *Ligustrum sempervirens *(Franch.) Lingelsh. are the most important incense species. Others serve as substitutes or are used to stretch incense powders.

**Conclusions:**

In Shaxi the use of incense mixtures at the household and community level is regularly practiced for communication with ancestors, ghosts, and deities and in some cases to strengthen self-awareness. Some of the documented species are widely used in central Asia and Europe, hinting at the well documented knowledge exchange that occurred in Shaxi, which was a major hub along the influential Southern Silk Road.

## Introduction

Ritual plants can be used in ritual healing [1], as hallucinogens [2], in incense or decorations for the communication with spiritual entities [3], or they can constitute sacred entities like trees [4]. The following focuses on incense plants, which are ritual plants used for their often fragrant smoke.

In comparison with the attention paid to the role of incense in religious symbolism, little effort has been made to study the ethnobotanical aspects of incense plants [5]. In Southeast Asia, Sangat-Roemantyo studied Javanese incenses, documenting the use of 14 plant species, and their production into different incense types [6]. Widjaja describes the use of *Dianella ensifolia *(L.) DC as incense in the context of Torajanese funeral ceremonies in South Central Celebes [7]. In the Himalayas, about 90 species are reportedly burnt as incense, of which different Junipers belong to the most important and frequently used [8]. Ritual uses of smoke of *Juniperus *spp. have been documented among ethnic groups of the Hunza valley (N Pakistan), the Ladakh Region (N India), the Manang District (Nepal), and among others [9-14]. In China there is a long history of the use of plant-derived smoke for hygiene, insecticidal, religious, warfare, and timekeeping reasons [15:134-154], and the combustion of incense during ceremonies and rituals has traditionally been an important aspect of Buddhist, Confucian, and Daoist religious practices. Many aromatic substances used for this purpose, such as frankincense, myrrh, and storax, were not produced domestically and were imported from African, European and other Asian countries [15:137]. A recently published compendium on the ethnobotany of plant-derived smoke documents a total of 14 different incense species used in China (of more than 400 species; [8]). Of these 14 species, five were originally documented in the Shuiluo Valley in Southwest China, where the Shuhi and other ethnic groups use ritual and incense plants for various religious purposes [3,16,17]. They select these plants according to their habitat and the color and smell of their smoke [6]. For the remaining incense species no details on their uses are available. Thus in contrast to the widely-known and globally traded incenses, little is known about the local use and variation among incense plants in China.

This paper therefore aims at documenting the use of incense plants in Shaxi, a remote valley in the Hengduan Mountains of Yunnan province in Southwest China, where previous ethnobotanical field research indicates the existence of ritual and incense plant knowledge.

## The study site

### Geographical aspects

**Figure 1:**
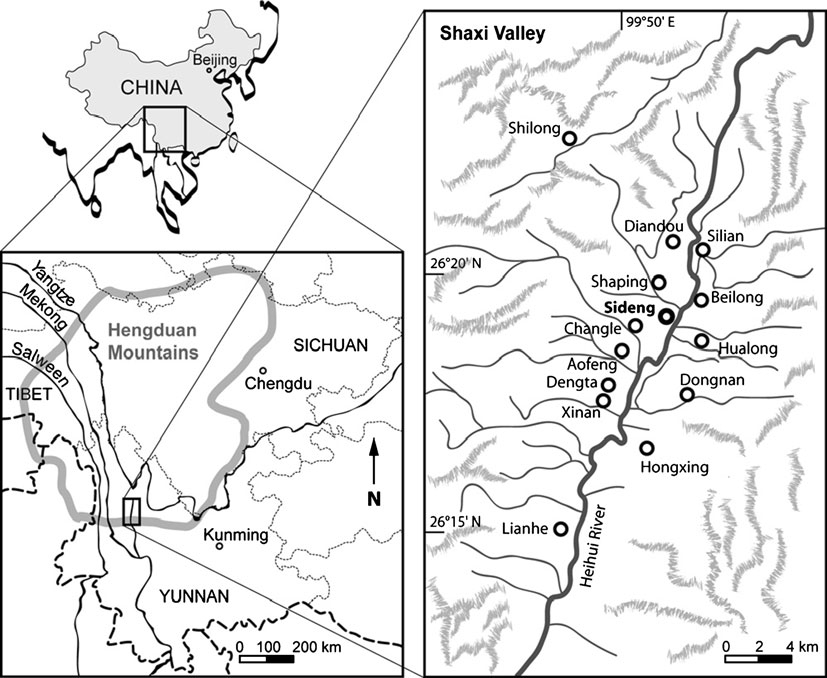
**Location of the study area in Southwest China, and the village groups of Shaxi (from Huber et al. 2010)**.

Shaxi Township (Jianchuan County, Dali Prefecture) lies in the Northwest of Yunnan Province, roughly between the two cities Dali and Lijiang (Figure 1). It belongs to the Hengduan mountain range, which is known for its rich biocultural diversity [18,19]. Shaxi encompasses an area of 288 km^2 ^and consists of a high plateau at an altitude of about 2100 m, which is flanked by two mountain chains that delimit the valley in the west and east and reach heights of up to 3100 m [20]. Heihui River, ultimately a Mekong tributary, flows through the fertile valley bottom in a north to south direction and is mostly enclosed by farmland and settlements. Agriculture is dominated by the cultivation of wet rice and tobacco in the valley bottom and corn, beans, and potatoes in the surrounding hills [21]. The adjacent mountains are largely vegetated with subtropical mixed pine (*Pinus yunnanensis *Franch.) and oak (*Quercus *spp.) forest [22].

Shaxi acted as the last stopover before the Southern Silk Road ascended to the Tibetan Plateau. It's elevated and interior geographical position is considered the main reason why it has remained relatively poor and underdeveloped. However, the lack of economical development is regarded as central for the survival of much of Shaxi's countryside and cultural heritage [23].

### Ethnographical aspects

In 2004, the township of Shaxi comprised approximately 23'000 inhabitants, of which the majority or 85.1% belong to the Bai ethnic minority. The Han people make up 11.2%, the Yi 2.4%, and 1.3% are part of the Lisu. While the Bai and Han typically live in the fertile valley bottom, members of the Yi and Lisu live in scattered settlements in the flanking western and eastern mountains [21].

As of 2000, the Bai population totaled 1.8 million members, the majority of which lived in China's southwestern provinces of Yunnan, Sichuan, and Guizhou [24:11]. In Yunnan, where "ethnic minorities" account for one-third of the total population, the Bai, after the Yi, form the second largest group comprising around 1.5 million residents [25]. The official classification by the People's Republic of China puts the Bai language close to the Yi branch in the Tibeto-Burman language family [26]. However, among linguists, still no consensus has been reached on the relationship of Baic to other languages (for a detailed discussion see [26]), since it contains elements similar to Tibeto-Burman, Chinese, and Thai and Mon-Khmer [27:p290].

### Religion

The term "China's Three Great Religions" is commonly used to refer collectively to Buddhism, Confucianism and Daoism and many temples throughout China are dedicated to all three religions. In Shaxi, statues depicting Confucian or Daoist gods or immortals can be observed in principally Buddhist temples or the other way around.

Ancestor worship is also practiced throughout rural China and is loosely associated with both Daoism and Confucianism. In the early 20th century, ancestor worship was practiced daily by the Bai people of Dali [28:94] and the importance of these practices in Shaxi are evidenced by the fact that in almost every household there is a shrine reserved for the worship of family ancestors.

The *Benzhu *Cult is often considered the religion of the Bai people and used as a defining character of this ethnic group. *Benzhu *has been referred to as "patron god worship", as many of the deities have a connection to local history, and each village has its own *Benzhu *in a specific temple, which is responsible for the village's safety [24:16]. Many of the *Benzhu*s of the Shaxi valley, however, have their origin in Buddhism and Daoism.

There are a number of locally worshipped deities in Shaxi, which are neither part of an institutionalized religion nor of the *Benzhu *Cult. *Tudigong *(Earth God) and his wife *Tudipo*, for example, are worshipped for matters related to agriculture, *Shanshen *(Mountain God) is worshipped before venturing into the hills, and *Longwang *(Dragon King) is worshipped for water related issues. Shrines dedicated to these local gods are omnipresent in the valley bottom as well as in agricultural fields in the lower hills, bridges, in the vicinity of water sources, and at the edges of villages. Additional deities are worshipped at home, such as the *Zaojun *(Kitchen God) and the *Menshen *(Gate Gods).

## Methods

The present study builds on our previous ethnobotanical studies in Shaxi, which started in 2005 [21,22]. Research focusing exclusively on ritual plant use and knowledge was conducted by the first author in September 2009 and May and June 2010. Additional information on the religious festivals in Shaxi was collected by the second author from June to December 2010.

Informants were chosen using the complementary strategies of snowball, purposive, and convenience sampling [29:186-194]. Informal conversation, unstructured, semi-structured, and structured interviews were carried out in Chinese with the help of an interpreter, who was familiar with the research area and fluent in English and Mandarin. All participants were fluent in Mandarin. Participant observation was undertaken to complement the interview data.

The interviews were carried out with a total of 44 Bai informants from Sideng (20), Changle (9), Shibaoshan (3), Silian (3), Diantou (2), Dongnan (2), Aofeng (1), Bailongtan (1), Shaping (1), Xinan (1), and Zongdeng (1). The average age of all informants was 56 years, 25 were female (50.3 ± 22.4 years), and 19 were male (64.1 ± 15.9 years).

The interviews covered questions on the topics of incense plant knowledge, uses, and associated beliefs. Structured interviews were carried out using representative parts of mentioned species that had been bagged in transparent plastic jackets. These were shown to the informants, who were then asked to tell the plant's name, its uses, the used parts, and how they are used. The local names of the species were recorded on tape and can be provided upon request. The recordings were performed with the help of three Bai persons of different ages and both sexes.

Use reports, listing the informant's name, species name, plant part used, and specific uses were compiled for the analysis of information about relevant plant species. All species were vouchered, and specimens were identified, following the nomenclature of the Flora of China [30], by the first author with the help of specialists at the herbarium of the Kunming Institute of Botany (KUN). Specimens are deposited at the herbaria of the Kunming Institute of Botany (KUN) and the University of Zurich (Z/ZT).

Research was conducted according to the Convention on Biological Diversity (CBD), including the Bonn guidelines on Access and Benefit-sharing (ABS). Field research took place in agreement with the responsible local authorities of Sideng and Kunming and in prior informed consent with all informants. To meet the fair and equitable Benefit-sharing guidelines, a set of labeled specimens together with summarizing descriptions of their uses were prepared to form part of an exhibition in the context of a medicinal plant garden project in Shaxi [31].

## Results and discussion

### Ritual plant knowledge in Shaxi

In total, 369 use reports of 20 plant species in 14 botanical families were documented. The collected information includes the names, the uses, and the habitats of the used species (Table 1). The species documented as ritual plants can be grouped into two main categories, namely those species burned as incense, and those which are used for the communication with spiritual entities without being burned (Table 2). Below we will mainly concentrate on the incense plants, i.e. the plants which are used for the smoke they produce.

**Table 1:** Documented ritual plants used among the Bai in Shaxi, Soutwest China

**Scientific name**	**Family**	Specimen number	**Habit**	**Habitat**	**Local names**^1^	**Parts used**	**Uses**^2^
*Artemisia *sp1	Asteraceae	100523-4/1 100530-1/1	Herb	Fields	bǎihāozǐ	Aerial parts, leaves	RIT, dried and rolled to balls to ignite incense; OTH, leaves bagged and hanged around the neck as scented sachet^3,4^, shoot axis dried used as chop sticks^3,4^, whole plant placed in rice field as pesticide^3,4^.
*Artemisia *sp2	Asteraceae	100528-1/1 100530-1/2	Herb	Fields	hēihāozǐ, qīnghāo	Shoot axis, aerial parts	RIT, dried shoot axis used as stick for joss stick production^4^; MED, aerial parts topically applied against inflammations^3,4 ^and to stop bleedings^3,4^; OTH, aerial parts steamed to clean air^4^, aerial parts rubbed to clean plates^3,4^.
*Basella alba *L.	Basellaceae	100523-1/1	Herb	Villages	téngqī	Subterranean parts	RIT, dried and powdered used as glue for joss stick production; CUL, subterranean parts edible^3,4^.
*Bambuseae*	Poaceae	100609-5/1	Shrub	Villages	zhúzi	Culm	RIT, dried, split culms used as sticks for joss stick production.
*Chamaecyparis obtusa *aff.	Cupressaceae	100530-1/4	Tree	Villages	biǎnbǎi	Branches, wood	RIT, dried and powdered branches and wood used as incense^3^; OTH, wood used to build coffins^3,4^.
*Cinnamomum glanduliferum *(Wall.) Meisn.	Lauraceae	100612-3/1 100607-2/1	Tree	Villages	xiāngzhāng	Leaves, fruits, wood	RIT, leaves dried and powdered used as incense^3^; CUL (MED), fruits edible, chewed against stomach ache^3^; OTH, wood is insect repellent therefore used to build containers for clothes^3,4^.
*Cornus oblonga *Wall.	Cornaceae	100529-3/1 100608-1/1	Shrub	Mountains	luceizi^4^, yiguizi^4^	Leaves, wood	RIT, leaves dried and powdered used as incense; FUE, wood used as firewood^3,4^.
*Cupressus funebris *Endl.	Cupressaceae	100523-3/1 100608-1/3	Tree	Villages	xiubai, sōngbǎi, xiāngbǎishù	Leaves, wood	RIT, leaves dried and powdered used as incense; wood chopped to match-sized pieces used as incense^4^; OTH, wood used to build coffins^3,4 ^and construct houses^3,4^, branches placed in rice fields as pesticide^3,4^.
*Gaultheria fragrantissima *Wall.	Ericaceae	100530-1/6 100619-1/1	Shrub	Mountains	fāngxiāngyóu	Leaves, fruits, aerial parts,	RIT, leaves dried and powdered used as incense; CUL, fruits eaten raw^3,4^; MED, decoction of leaves used for postnatal washing^3,4^; OTH, leaves steam distilled to produce fragrant liquid^4^, aerial parts placed in rice fields as pesticide^4^.
*Gonostegia hirta *(Blume) Miq.	Urticaceae	100601-1/1	Herb	Villages	mùjué, nuòmǐcǎo^4^	Subterranean parts	RIT, dried and powdered used as glue for joss stick production.
*Imperata cylindrica *(L.) Rausch.	Poaceae	100527-1/1 100608-1/5 100609-1/2	-	Fields, Mountains	zǐsūncǎo	Whole plant	RIT, planted on top of graves to support prosperous family development.
*Juniperus formosana *Hayata	Cupressaceae	100530-1/3	Tree	Villages	cisong^4^, cìbǎi, shānshù^4^	Leaves	RIT, leaves dried and powdered used as incense.
*Juniperus squamata *Buch.-Ham.	Cupressaceae	100530-1/5	Tree	Villages	cìbǎi, shānshù^4^	Leaves, wood	RIT, leaves dried and powdered used as incense^3^, wood chopped to match-sized pieces used as incense^4^.
*Ligustrum sempervirens *(Franch.) Lingelsh.	Oleaceae	100529-2/1 100602-1/1 100608-1/4 100622-1/1	Shrub	Mountains	dongbuxiu, waibeizi^4^, dòubànxiāng, xiāngyè	Leaves	RIT, dried and powdered used as incense.
*Lyonia ovalifolia *(Wall.) Drude	Ericaceae	100609-4/2	Shrub	Mountains	xiumulu^4^, geizimeimeixi^4^	Leaves	RIT, dried and powdered used as incense^3,4^.
*Pinus yunnanensis *Franch.	Pinaceae	100529-4/1	Tree	Mountains	sōngshù, sōngbǎi^4^	Root	RIT, dried and powdered used as incense, used as fire powder on Torch Festival^3^.
*Pistacia weinmanniifolia *J. Poiss. ex Franch.	Anacardiaceae	100609-2/1	Shrub	Mountains	aixiang^4^	Branches	RIT, dried and powdered as incense.
*Populus *sp.	Salicaceae	100608-1/2	Tree	Mountains	baizou, baizong, báihuà^4^, báichá^4^, báiyángshù^4^	Branches	RIT, inserted into holes of gates and walls to prevent evil spirits and small animals from entering.
*Prinsepia utilis *Royle	Rosaceae	100529-1/1	Shrub	Mountains	z(h)ongdaqi, qīngcìguǒ, qīngpíshù^4^	Branches, fruits, seeds	RIT, branches inserted into holes of gates and walls to prevent evil spirits from entering; CUL, fruits collected and pressed for oil production, young leaves edible^3,4^; MED, seed-oil rubbed on skin against-inflammations^3,4^.
*Ternstroemia gymnanthera *(Wight & Arn.) Bedd.	Theaceae	100609-4/1	Shrub	Mountains	azijixiu^4^, xiangye	Leaves, aerial parts	RIT, leaves dried and powdered used as incense^3^; OTH, aerial parts placed in rice fields as pesticide^4^.

The specimens were collected and identified by the first author.

^1^Local names are transcribed using Hanyu Pinyin without tones for Bai names, and with tones for Mandarin names.

^2^CUL, culinary use; FUE, fuelwood; MED, medicinal use; OTH, other uses; RIT, ritual use.

^3^Use not observed during field work.

^4^Information mentioned by less than three informants.

**Table 2:** Ritual plant categories based on interview and observation data

Use categories	Species	Parts used
**Ritual plants**		
Incense		
*Burned in censer*		
Powders	*Chamaecyparis obtusa *aff.	Branches
	*Cinnamomum glanduliferum*	Leaves
	*Cornus oblonga*	Leaves
	*Cupressus funebris*	Leaves
	*Gaultheria fragrantissima*	Leaves
	*Juniperus formosana*	Leaves
	*Juniperus squamata*	Leaves
	*Ligustrum sempervirens*	Leaves
	*Lyonia ovalifolia*	Leaves
	*Pistacia weinmanniifolia*	Branches
	*Ternstroemia gymnanthera*	Leaves
Woods	*Cupressus funebris*	Wood
	*Juniperus squamata*	Wood
Combustible agent	*Artemisia *sp1	Aerial parts
*Joss stick production*		
Sticks	*Artemisia *sp2	Shoot axis
	Bambuseae spp.	Shoot axis
Glues	*Basella alba*	Subterr. parts
	*Gonostegia hirta*	Subterr. parts
Powders	*Cupressus funebris*	Leaves
	*Pistacia weinmanniifolia*	Branches
	*Ternstroemia gymnanthera*	Leaves
Combustible agent	*Pinus yunnanensis*	Root
		
Ghost plants	*Populus *sp.	Branches
	*Prinsepia utilis*	Branches
Other	*Imperata cylindrica*	Whole plant

**Figure 2:**
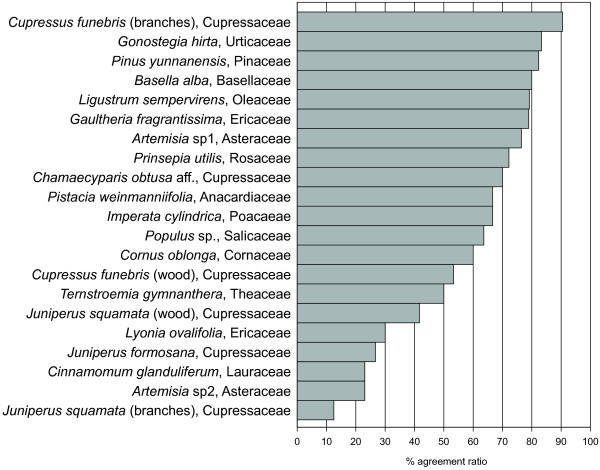
**Agreement ratios of the informants on ritual plant species with five or more use reports**. Agreement ratios are calculated as the percentage of the number of informants who identified a specific species as ritual plant divided by the number of informants totally asked.

Knowledge of specific ritual plants differed considerably among the informants. To quantify the degree of agreement among the informants on whether specific species are used as ritual plants, an agreement ratio was calculated for all species with five or more use reports (Figure 2). Overall, 15 species show an agreement of 50% or more. *Cupressus funebris *Endl. is the species with the highest agreement ratio (90%), indicating that most of the informants agreed on its use as a ritual plant. On the contrary, the use of *Cinnamomum glanduliferum *(Wall.) Meisn. (23%) as ritual plant is controversial, as some informants questioned its ritual use.

### Incense plants

A total of 17 species are used as incense and for joss stick production. They belong to 12 different families, 4 species in the Cupressaceae family, and comprise shrubs (7 spp.), trees (6 spp.), and herbs (4 spp.). The parts of the plant used are the leaves (9 spp.), branches (2 spp.), tubers (2 spp.), wood (2 spp.), shoot axes (2 spp.), roots (1 sp.), and aerial parts (1 sp.). The species are always used as mixtures and are either burned in the form of powders in a censer or as joss sticks. According to most informants, the smell of the smoke is the main criterion for the selection of incense plants.

#### Incense powders burned in a censer

**Figure 3:**
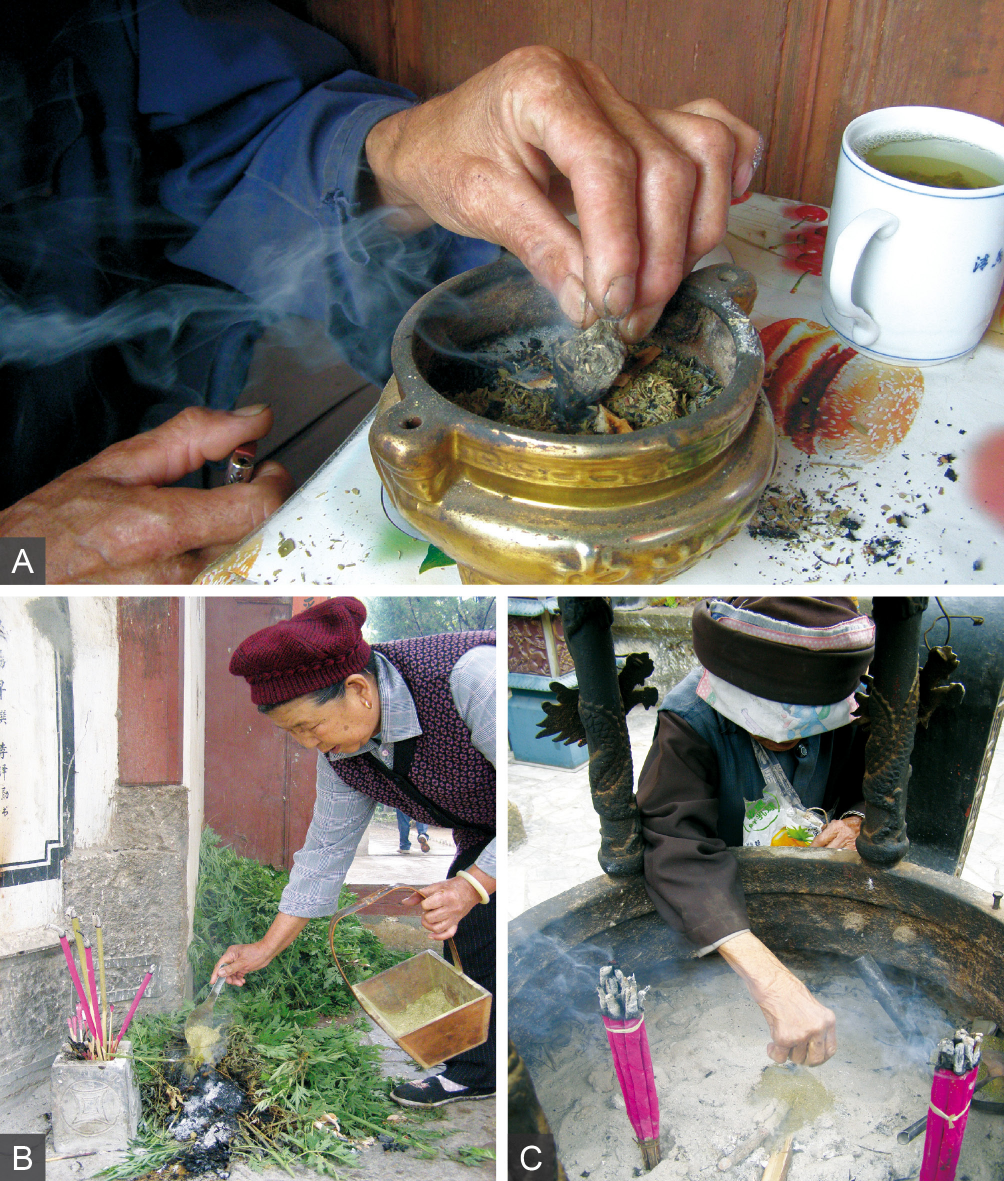
**Incense plant uses. A,** Incense is burned in a small censer. Clumped aerial parts of *Artemisia *sp. ignite both powder and wood. **B**, *Benzhu *temple entrance in Sideng. Bai woman spoons incense from a wooden basket on to a smoldering heap of *Artemisia argyi*. **C**, Large incense burner in the Haiyun temple courtyard. Bai woman adding incense powder to smoldering *Cupressus funebris *branch (all by Staub 2010).

For the burning of incense powder in a censer, three small match-sized pieces of *Juniperus squamata *Buch.-Ham. or *Cupressus funebris *wood are placed on an incense heap. Then, a dried ball of *Artemisia *sp1 is lit and added to ignite both powder and wood (Figure 3A). It was mentioned that *Cupressus funebris *wood is easily available and therefore more widely used, whereas that of *Juniperus squamata *has the better smell and is only used on special occasions. It was further explained by a Buddhist nun that *Juniperus squamata *is more widely used in Tibetan areas, where, according to her, in general, better smelling incense is used. Her statement fits the observations in various temples throughout Shaxi. While incense powder, *Artemisia *sp1, and *Cupressus funebris *wood were freely available in almost all surveyed temples, the more exclusive wood of *Juniperus squamata *was only found in the uppermost temple of Shibaoshan, an ancient monastery above the Shaxi valley. In such very well equipped temples, incense powders are usually stored in wooden baskets together with wooden spoons, which help to make use of the powder (Figure 3B). In more simply furnished temples, the same materials are stored in plastic containers.

While smaller censers are usually found in the prayer halls of temples, the larger, head-high incense burners are placed in the middle of temple courtyards (Figure 3C). In these burners, larger amounts of incense are burned with branches of *Cupressus funebris *and without *Artemisia *sp1.

#### Incense burned as joss sticks

**Figure 4:**
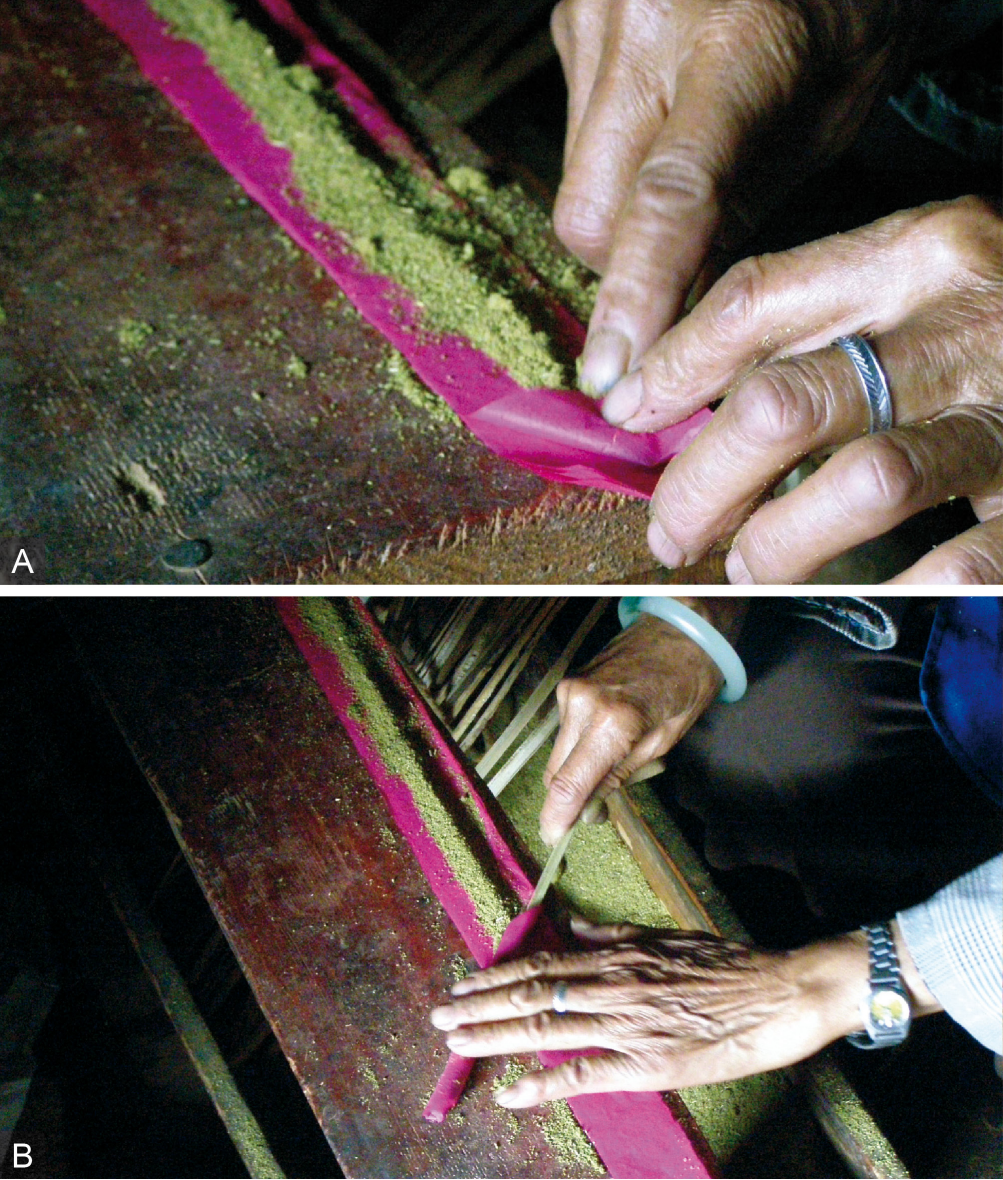
**Local production of red joss sticks.** A, B, For red joss-stick production, cotton paper is folded over a stick, and wrapped around it (both by Staub 2010).

During field research, the production of incense sticks was observed on four occasions. The producers were Bai women between the ages of 54 and 80 years, and they produced the sticks in similar ways. Two kinds of sticks, red and green ones, are locally produced, traded, and used. For the production of red sticks, red cotton paper is laid out on a solid base and fixed on one side with a needle or a weight. Then, incense powder is evenly distributed over the paper. A wooden stick of about 50-55 cm length, made of split bamboo culm, a shoot axis of *Artemisia *sp2, or other available wood, is held against the paper at an angle of ca. 30 degrees. The tip of the paper is folded over the stick, closing the upper end of the incense stick (Figure 4A). Then, the stick is thoroughly rolled over the paper until the entire paper is twisted around (Figure 4B). The remaining free tip of the paper is then glued onto the stick with an adhesive consisting of water-solved wheat flour.

**Figure 5:**
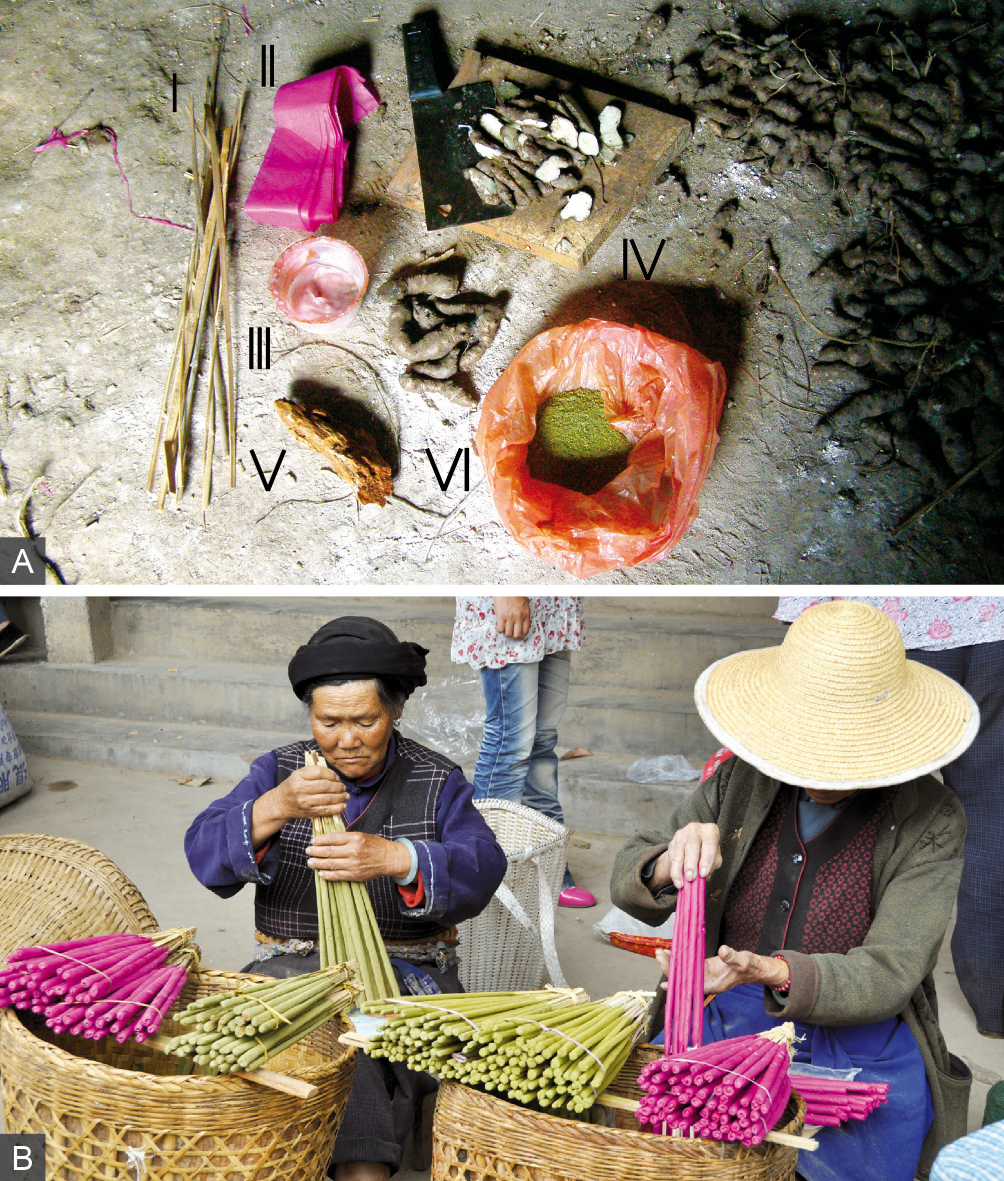
**Joss stick production and trade. A,** The materials for the production of red joss sticks comprise dry shoot axis (I), red cotton paper (II), wheat flour glue (III), sticky tubers (IV), dry *Pinus yunnanensis *root (V), and incense powder (VI; by Staub 2010). **B**, Joss-stick sale at the local weekly market in Sideng (by Ehrlert 2010).

For the production of green sticks, glue instead of paper is used to attach the incense powder to the stick. The same kinds of wooden sticks are first dipped in water and then in a plant powder that makes the surface adhesive. The plant powders used are made of sliced, dried, and later powdered subterranean parts of either *Gonostegia hirta *(Blume) Miq. or *Basella alba *L. (Figure 5A). *Gonostegia hirta *is formerly a cultivated species that can now be found in abandoned fields and is preferred over the weedy growing *Basella alba *because of its better adhesive quality. After becoming gluey, the sticks are again dipped in water and covered with dried root powder of *Pinus yunnanensis*, which improves combustibility. Once again the sticks are dipped into water and covered with incense powder. The procedure of adhering incense powder onto the sticks is repeated until the sticks are of the desired thickness. Finally, the sticks are kneaded to straighten their shape and left to dry. The red and green sticks are sold by the producers for approximately 1 Yuan (= 0.12 USD at the time of field work) per bunch of 20 sticks at the local weekly market (Figure 5B). The plant material is usually collected by the producers themselves in the surrounding fields and mountains.

#### Species composition of incense powders

Eight different powders for censer use and three different ones for joss-stick production were collected from temples, incense users, and producers. Table 3 shows the species composition of the powders as mentioned by the informants and verified microscopically. In all but one powder, *Cupressus funebris *is present, suggesting its role as basic ingredient of the mixtures. Regarding powders for censer use, *Gaultheria fragrantissima *Wall. and *Ligustrum sempervirens *Lingelsh. are also frequent components. The central role of these three species is also supported by interview statements and their high agreement ratio (Figure 2).

**Table 3:** Compositions of the collected incense mixtures

	Mixture number										
Powder constituent	C1	C2	C3	C4	C5	C6	C7	C8	S1	S2	S3
*Cupressus funebris*	X	Y	X	X	X		Y	X	X	X	X
	
*Ligustrum sempervirens*		Y	X	Y			Y				
	
*Gaultheria fragrantissima*	X		X			X					
	
*Pistacia weinmanniifolia*											X
	
*Ternstroemia gymnanthera*											X

C Mixture for censer use

S Mixture for joss stick production

X Constituent mentioned by informant

Y Constituent identified by the first author

Both the joss-stick and the censer-use mixture compositions vary. Although eleven species were named to be used as incense, only five were mentioned as compounds of the collected incense powders. Why the remaining six species do not occur in the mixtures is uncertain. Possible explanations are that the informants who provided the mixtures did not know the exact composition, or that they pretended not to know in order to keep the details of their mixtures secret. Other potential explanations are that species which are not present in the mixtures are of facultative use (i.e., if other species are not available) or that they are used intentionally to stretch incense. Opinions among informants differed on this issue. It was mentioned that *Cornus oblonga *Wall., *Ternstroemia gymnanthera *(Wight & Arn.) Bedd., *Lyonia ovalifolia *(Wall.) Drude, *Ligustrum sempervirens*, and *Cinnamomum glanduliferum *can indeed be used to stretch or fake the powders. However, it was also claimed that because of increasing pressure resulting from the large number of incense producers, people were compelled to use the materials that are available. As a result of this pressure, there are many species combinations.

### Contexts of incense use

The contexts of the documented incense uses are diverse and include aspects of meaning, purpose, time, and place. In interviews, informants mentioned various reasons for burning incense. Usually, incense is burned to communicate with spiritual entities and for fumigations that enhance personal well-being. The communicative act with spiritual entities was the most frequently mentioned reason for fumigations. The locations where incense is burned for these communicative acts include graves, shrines, temples, and kitchen stoves. Each place is connected to a unique myth about the spirits that can be called there. These places and spirits are described below.

In almost all documented contexts, it was not the type of incense burned that was deemed to be significant but rather that it was burned at all. Although no fundamental difference between sticks and powders was found, certain differences in their practical functionality were observed. Sticks were usually preferred for outside use because they are less affected by weather and because expensive censers are not needed. On the contrary, powders tend to be used inside houses and temples, where the necessary censers are available.

#### Fumigations inside the house

The household use of incense takes place on a daily or periodical basis, usually in the form of burning incense sticks. The timing of fumigations varies, and can take place in the morning before breakfast, at noon before lunch, and in the afternoon. During the fieldwork, fumigations were never observed in the evening. Typical days of usage are traditional holidays like the *Guijie *(Ghost Festival), *Chunjie *(Spring Festival), *Duanwujie *(Dragonboat Festival), *Qingmingjie *(Tomb Sweeping Day), and *Jiuyuejiu *(Double Ninth Festival). For a detailed description of the festivals and the plants used see Appendix 1.

The most conspicuous place for incense burning is at the entrance gates of a house, where incense sticks are placed in a special holder fastened to one side of the gate so as to prevent bad influences from entering the house and the courtyard. Incense is also burned on a small altar close to the stove in the kitchen of a house where *Zaojun *(Kitchen God), can be worshiped. It was mentioned that the same altar is also a place for ancestors to meet the household members and to enjoy a meal. Incense is thus burned to invite the ancestors into the home.

#### Fumigations at temples

Communal incense use usually takes place on special occasions, such as ceremonies in temples. Both incense sticks and powders are used. Powders are predominately used inside temples, where the necessary censers and incense burners are available. Sticks are used outside as well as inside the temples.

#### Fumigations at trees, wells, and for the mountain gods

**Figure 6:**
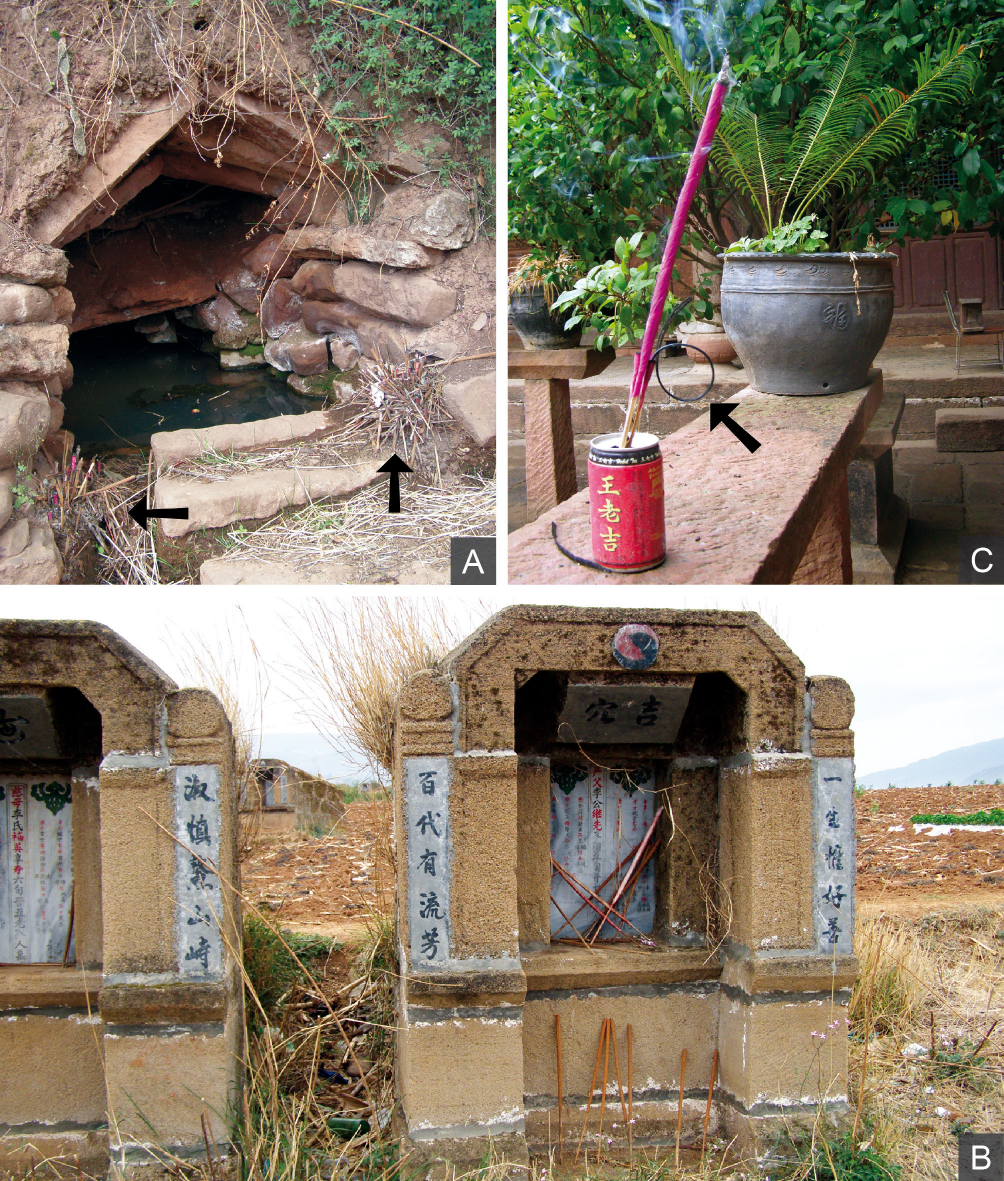
**Incense plant use sites. A,** Remains of past incense use at a well (indicated by arrows). **B**, Joss stick offerings at grave sites are left unburned due to wildfire hazard. **C**, Joss sticks in a can. The curled shape of the burned stick is a symbol of luck (indicated by arrow; all by Staub 2010).

Burnt and unburnt joss sticks were observed at the sites of large and ancient trees. It was explained that they are placed there to worship *Zhengci *(local Tree God). Incense is also burned at water wells, where it can be used to show respect to *Longwang *(Dragon King; Figure 6A). Also, other gods such as *Guanyin *(Goddess of Mercy) are worshiped in special places outside the villages. These deities are preferably called for blessings before departure to or after returning from the mountains.

#### Fumigations at graves

Graves are another important site for the use of incense sticks. Due to the danger of wildfires, the sticks are not burned, but only placed on the graves (Figure 6B). It was mentioned that there is a series of dates on which incense should be brought to the graves by the relatives of deceased persons. These dates are, one day, three days, one week, three weeks, and 100 days after the person died, and additionally every 1st and 5th of the sixth month of the lunisolar calendar (LC), and on *Qingmingjie *(Tomb Sweeping Day).

#### Fumigations on weddings

Weddings are another occasion during which fumigations are performed. On such auspicious occasions incense sticks made of bamboo, which if produced properly will curl with combustion, are preferred (Figure 6C). Curling of the stick, a desirable symbol of luck, occurs when the bark of the bamboo culm has been peeled correctly.

#### Fumigations for well-being

Some informants mentioned the influence that fumigations have on the well-being of the user. For example, it was explained that fumigations in combination with the chanting of certain verses and prostrations can be performed by women to heal sick family members. These actions are seen as an offering to bad, old ghosts that cause diseases and can be driven out this way. Other informants described the linkages of fumigations and personal well-being through more psychological aspects. Some feel that people who burn incense just feel happier, which in turn can improve personal health. A Buddhist nun in one of the temples underlined, that incense has mainly an effect on the individual performing the fumigation, i.e. as a way to strengthen self-awareness:

"Burning incense has five main effects:

- It is a statement of not doing bad things any more.

- Through the burning of incense you settle down.

- Through the burning of incense you get rid of hardness.

- Burning incense increases wisdom.

- Through the burning of incense you get rid of the feelings about precious old things." (Buddhist nun; 55 y) - Fieldnotes, May, 2010

Several informants ascribed pharmacological activity to the smoke of incense: For example, the effect of incense smoke to clean the air, especially in crowded rooms like temple halls, and in that sense of killing germs, was mentioned.

Besides the general consent that the fumigations practiced in Shaxi are traditionally linked with religion, some informants dismissed them as superstition:

"I don't burn incense by myself and think that young people in general have little time to burn incense. The young people don't believe in superstition and don't belong to a religion. Nonetheless, I feel comfortable when smelling it. In my family, my mother burns incense and, in general, older women do it. They are all victims of superstition [*mixin*]. The younger generation has experienced another education than the older women, so they do not believe in Buddhism anymore." (F, 23 y) - Fieldnotes, May, 2010

### Other ritual plants

**Figure 7:**
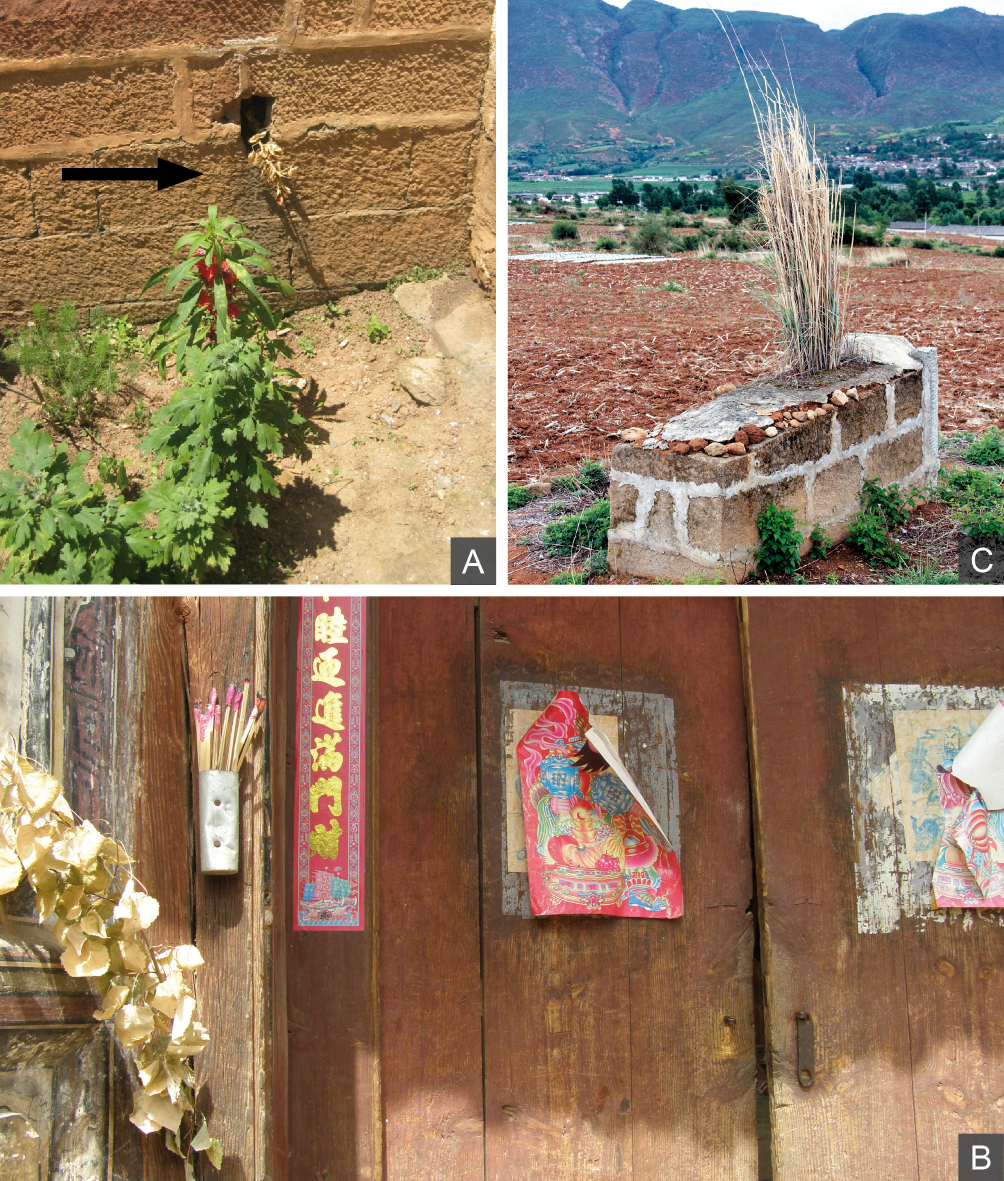
**Ritual plant use sites. A,***Prinsepia utilis *branch stuck into the water drain of a house to ward off ghosts (indicated by arrow; by Staub 2010). **B**, Incense and *Populus *sp. branch are placed in a holder at one side of the entrance of a house (by Hess 2010). **C**, A grass species was planted on top of a grave to support the prosperous development of the family (by Hess 2010).

*Prinsepia utilis *Royle and *Populus *sp. can be placed in the category of "ghost plants". The uses of both species to ward off evil spirits, ghosts, or bad things were mentioned by many informants, and both species were observed to hang on many walls and gates of houses in Shaxi. Both species occasionally occur in the eastern mountains, where they are collected for use. *Prinsepia utilis *branches are stuck into cracks in gates or house walls mostly on the 1st day of the 7th month (LC; Figure 7A). This use of *P. utilis *is connected to *Guijie *(Ghost Festival), which takes place on the 14th the same month (LC; see also Appendix 1**)**. The branches of *P. utilis *prevent the ghosts that are released from heaven between the 1st and the 15th day of the 7th month (LC) from entering the home. Similarly, branches of *Populus *sp. were often observed to be stuck into house gates or holes of outer house walls (Figure 7B). *Populus *sp. branches are hung to keep malevolent spirits outside. Other uses of these branches include being hung at the beginning of summer to prevent snakes and small animals from entering, and by attaching to entrance gates on the 30th of the sixth month (LC) to keep unwelcome people outside.

The use of *Imperata cylindrica *(L.) Rausch. was directly observed on the occasion of a funeral; then it was planted on top of the grave directly after the burial of the deceased person. On top of a grave this is believed to support the prosperous development of the family, especially if it has grown well and developed many stolons. The use and meaning of this species are also reflected in its local name, *Zisuncao*, meaning the child's and grandchild's grass. The planting of *Imperata cylindrica *and other grass species on top of graves seems to be a widespread practice that was observed in many villages of Shaxi (Figure 7C).

### Comparison with ritual plant uses outside Shaxi

Studies from the Shuiluo valley in the neighboring province of Sichuan also describe the use of *Cupressus funebris, Pistacia weinmanniifolia *Poiss. ex Franch., *Juniperus squamata*, and *Cornus oblonga *as incense [3] (Weckerle and Bühler, unpublished data). In Shaxi, however, these species are burnt dry, powdered, and mixed, whereas in Shuiluo they are applied fresh or dried, whereby the whole branches are commonly used, rather than making mixtures. In addition, all of these species are used both as incense and as decoration to invite deities in Shuiluo, while their ritual use is limited to incense in Shaxi. Although *Pinus yunnanensis *is used as incense both in Shaxi and Shuiluo, their uses are very dissimilar. While the root is used as side material for joss stick production in Shaxi, its branches are burned as incense in Shuiluo. In Shuiluo, incense species are selected for their habitat as well as the smell and the color of their smoke, whereas in Shaxi, the smell seems to be the only important factor.

Besides these striking similarities of ritual plant uses on a species level between the Shaxi and Shuiluo valley, comparable uses from other areas also exist on the genus level for the genera *Artemisia, Cinnamomum, Cupressus, Juniperus *and *Pistacia *[8]. The burning of *Juniperus *spp. as incense is a widespread practice in the whole Himalayan region and beyond [8,32]. For example, the Hunzakut of the Hunza valley (NW Pakistan) burn Junipers during rituals in which ritual specialists communicate with the supernatural world [9]. Also *Artemisia *spp. have reportedly been used as incense in the Himalayas, in particular Nepal and India, [10,13,33]. In the Lahoul valley (NW India), members of the Beeling tribe even burned *Artemisia maritima *L. var. seski together with different Junipers [34]. The use of *Cinnamomum *species for incense purposes is also documented from Indonesia (*C*. *burmannii *(Nees & T. Nees) Blume and *C*. *sintok *Blume), India (*C*. *camphora *(L.) J. Presl), and the Himalayas (*C*. *tamala *T. Nees & Eberm. and *C*. *verum *J. Presl) [8]. Additionally, fumigations with *Cupressus torulosa *D. Don have been reported from Nepal and such with *Pistacia lentiscus *L. from Vietnam [8]. No information was found on the use of the remaining species as incense or for fumigations.

## Conclusions

The Bai people of Shaxi use a variety of ritual plants, especially incense plants, both on a communal and household level. The documented incense plants were never used solely, but only in mixed powders or in the form of joss sticks. Ritual fumigations are mainly conducted for the communication with ancestors, spirits, and deities, and in some cases also for personal well-being and to strengthen self-awareness. Some informants mentioned pharmacological effects, and some dismissed the fumigations practiced by others as superstition. Incense use was documented in diverse contexts. Among these contexts, no significant differences regarding the type of incense used, but differences in the contexts of place, time, and meaning of the uses were found. The foremost quality for which species are selected as incense seems to be the smell of their smoke. Interview data and the incense powder compositions strongly suggest that *Cupressus funebris, Gaultheria fragrantissima*, and *Ligustrum sempervirens *are the central incense species. Possible roles of the other species range from being used to stretch incense powders to serving as substitutes for other, unavailable species.

Similar uses of *Cupressus funebris, Pistacia weinmanniifolia, Juniperus squamata*, and *Cornus oblonga *were also reported from the Shuiluo Valley in the Hengduan mountain range. *Juniperus squamata *is used as incense in even wider parts of the Himalayas. These finding indicate that the documented incense plant knowledge probably does not constitute exclusive knowledge of the inhabitants of Shaxi and may be strongly influenced by Tibetan Buddhism and the knowledge exchange along the Southern Silk Road, which has been influential to Shaxi for hundreds of years.

## Competing interests

The authors declare that they have no competing interests.

## Authors' contributions

The first and last authors designed the study, drafted and finalized the manuscript. POS and MSG conducted field research and CSW supported both during the research project. All three authors read and approved the final manuscript.

## Appendix 1

### Important festivals in Shaxi

Ten local informants were asked to list and describe the most important festivals throughout the year. They were also asked if plants play an important role for the practices on these festival days. If not otherwise stated, plants do not play an important role, except for incense, which is burned on all of these occasions. The presented dates follow the Chinese lunisolar calendar.

#### Chunjie (01.01)

The Spring Festival or Chinese New Year marks the beginning of the new lunar year. For many people in Shaxi it is the most important festival throughout the year. Family members working outside the valley usually return home for the festivities. The celebrations last for one week, feasts are common and firecrackers are let off throughout the whole week. On the New Year's Dinner, almost all families eat fish, as the Chinese word for fish (*yu*) is pronounced the same way as the word for surplus. Paper-cuts in red displaying the character for good fortune (*fu*) are hung on doors and windows. Usually, at least one member of each family goes worshipping at the Chenghuang temple, to ask for the prosperous development of the family in the new year.

#### Taizihui (08.02.)

The Prince Festival is celebrated to remember the historical Buddha (*Shakyamuni*) as he was still Prince Siddhartha Gautama, wandering through his father's lands. On this day, a Buddha statue from the Three Religions Temple in Changle is put on a palanquin and carried to the Xingjiao temple in Sideng. Thereafter, a statue is carried throughout the village and the party holds at every gate, so the prince can bless the household. Local boys are dressed up as the Prince Siddhartha and people from throughout the valley participate in the parade. This festival is only celebrated by some Bai communities, particularly in the counties of Jianchuan and Eryuan. None of the local people knew where the tradition originated.

#### Qingmingjie (106th day after *dongzhi *(winter solstice))

The Tomb Sweeping Day is celebrated to commemorate the family's ancestors. Usually the whole family sets out for a picnic at the family's gravesite. Incense is burnt, some *sutras *sung and fresh willow branches are stuck on the graves. Children often use willow branches to create «crowns», which they wear on their head. The history behind the festival or the usage of willow branches was not known to any local people questioned.

#### Sanyuesan (03.03.)

All informants mentioned that this and related festivals (*Liuyueliu *and *Jiuyuejiu*) are Buddhist festivals celebrated because of the symbolic value of the numerals of the dates. No one considered these to be important festivals in Shaxi. Nonetheless, all three are celebrated by large crowds, and *Mamahuis *(religious women's associations) from distant villages use these opportunities to visit one another. They are, however, not celebrated in families but only in temples, where the *Mamahui*s hold ceremonial dances and prepare food and paper figures for offerings.

#### Duanwujie (05.05)

The Dragonboat Festival is celebrated in Shaxi in memory of a legend, in which two beautiful girls are turned into a blue and a white snake. On this day, the evil spirits symbolized by these snakes come to haunt the living. In order to protect the family from the negative influence poplar (*Populus *sp.) twigs are stuck in the corners of the home and close to openings (doors, windows, crevices etc.) in the walls of the courtyard. These are supposed to hinder the ghosts from entering. On this day *Zongzi *(glutinous rice stuffed with various fillings and wrapped in bamboo leaves) are prepared and eaten. Further, a special soup is prepared of local herbs and drunken as a tonic by the festival participants.

#### Liuyueliu (06.06)

see *Sanyuesan*

#### Huobaojie (25.06.)

The Torch Festival is traditionally only celebrated by the Bai and Yi people to commemorate *Baijie Wangfu*, the wife of a historic regional king. On the festival day, on the central square of each Bai village a decorated pole is erected, on which pieces of wood are fixed in order to form a large torch. This is set on fire in the evening after traditional dances have been held. Afterwards, boys and young men walk around with a small torch and a sack full of powder, prepared from half-rotten pine stumps. The torch is held towards the legs of other people and the powder is thrown in the flame, producing a large darting flame. This is supposed to bring good luck to the «burned». Every woman, who had given birth to a child in the previous year - or a close female relative -, must walk around throughout the evening providing rice liquor, candy and sunflower seeds to the visitors. The more people take what she supplies, the more prosperous will the newborn child's development be.

#### Guijie (14.07.)

The Ghost Festival actually marks the end of a two weeks period, during which the ghosts - both good and evil - were free to roam the world of the living. On the first of the lunar July, all souls are released from the underworld. Before this day, virtually all families stick branches of *Prinsepia utilis *Royle close to all openings of the courtyard to keep the ghosts outside of the home. As young children are reportedly particularly susceptible to the ghosts' influences, they are not allowed to leave the house after nightfall during the next two weeks.

On the sunset of the 14th, all ghosts are called back to the underworld. In order to provide for the needs of the ancestors' souls, all families burn incense and special paper money and paper clothes. There are different clothes for deceased children, women and men. All of them have the respective ancestors name inscribed, which is read out loud by the head of the family when put into the flames. Further, a large meal is prepared and parts of each dish are also put into the flames to let the ancestors participate in the feast.

#### Zhongqiujie (15.08.)

The Mid Autumn Festival is celebrated with a large feast where all family members should be present. Afterwards, a table with apples, chestnuts, pears, walnuts, water chestnuts (*Eleocharis *dulcis Trin. ex Henschel.), other snacks and most importantly *yuebing *(mooncakes) is placed outside and the food is eaten while marveling at the full moon.

#### Jiuyuejiu (09.09.)

see *Sanyuesan*

#### Dongzhijie (At the day of winter solstice)

The Winter Solstice Festival is celebrated with the immediate family to commemorate the shortest day of the year. The only fixed ritual for this festival is to make *tangyuan *(sweet dumplings). Usually, the whole family helps to prepare the filling and then forms the dumplings out of dough made of glutinous rice.
